# Stratification of Candidates for Induction Chemotherapy in Stage III-IV Nasopharyngeal Carcinoma: A Large Cohort Study Based on a Comprehensive Prognostic Model

**DOI:** 10.3389/fonc.2020.00255

**Published:** 2020-02-28

**Authors:** Xue-Song Sun, Bei-Bei Xiao, Zi-Jian Lu, Sai-Lan Liu, Qiu-Yan Chen, Li Yuan, Lin-Quan Tang, Hai-Qiang Mai

**Affiliations:** ^1^Sun Yat-sen University Cancer Center, State Key Laboratory of Oncology in South China, Collaborative Innovation Center for Cancer Medicine, Guangdong Key Laboratory of Nasopharyngeal Carcinoma Diagnosis and Therapy, Guangzhou, China; ^2^Department of Nasopharyngeal Carcinoma, Sun Yat-sen University Cancer Center, Guangzhou, China

**Keywords:** nasopharyngeal carcinoma, Epstein–Barr virus DNA, induction chemotherapy, radiotherapy, survival

## Abstract

**Objective:** To establish a prognostic index (PI) for patients with stage III-IV nasopharyngeal carcinoma (NPC) patients to personalize recommendations for induction chemotherapy (IC) before intensity-modulated radiotherapy (IMRT).

**Patients and Methods:** Patients received concurrent chemoradiotherapy (CCRT) with or without IC. Factors used to construct the PI were selected by a multivariate analysis of progression-free survival (PFS), which was the primary endpoint (*P* < 0.05). Five variables were selected based on a backward procedure in a Cox proportional hazards model: gender, T stage, N stage, lactate dehydrogenase (LDH), and Epstein–Barr virus (EBV) DNA. The cutoff value for the PI was determined by the receiver operating characteristic curve analysis.

**Results:** The present study involved 3,586 patients diagnosed with stage III-IV NPC. The cutoff value for PI was 0.8. The high-risk subgroup showed worse outcomes than did the low-risk subgroup on all endpoints: PFS, overall survival (OS), locoregional relapse-free survival (LRFS), and distant metastasis-free survival (DMFS). In the low-risk subgroup (PI <0.8), patients showed comparable survival outcomes on all clinical endpoints regardless of IC application, whereas in the high-risk subgroup (PI > 0.8), the addition of IC significantly improved PFS, OS, and DMFS, but not LRFS. In multivariate analyses, IC was a protective factor for PFS, OS, and DMFS in the high-risk subgroup, while it had no significant benefit in the low-risk subgroup.

**Conclusion:** The proposed prognostic model effectively stratifies patients with stage III-IV NPC. High-risk patients are candidates for IC before CCRT, while low-risk patients are unlikely to benefit from it.

## Introduction

Nasopharyngeal carcinoma (NPC), a malignant disease of the nasopharyngeal epithelium, has an incidence rate of 20–50 cases per 100,000 people in epidemic areas, such as Southeast Asia (1, 2). As NPC has high sensitivity to irradiation and a distinct anatomical location, radiotherapy (RT) is currently the only curative treatment for NPC. For stage I NPC, radiotherapy has been reported to achieve an overall survival (OS) rate of over 90%. For locoregionally advanced disease, which represents 70–90% of newly diagnosed NPC cases, radiotherapy with concurrent chemoradiotherapy (CCRT) could improve OS; thus, it is regarded as standard treatment (3–5). However, over 20% of patients still develop distant metastasis after CCRT (6).

By lowering tumor volume and restricting occult micrometastasis, induction chemotherapy (IC) before RT has been proposed to further decrease distant metastasis risk. Previous studies have shown that IC might improve survival outcomes in patients with locoregionally advanced NPC (7, 8). Nevertheless, not all patients with locoregionally advanced NPC benefit from IC. Previous studies have reported that IC might not improve survival among patients with T3-4N0-1 NPC; in fact, IC has been associated with severe toxicity in this patient group (9, 10). Given the body of evidence, it is likely that patients at high risk might benefit from IC more than patients at low risk. As such, a prognostic score to differentiate high- and low-risk patients is required to assist in clinical decision-making.

The tumor, node, metastasis (TNM) staging system, which continues to be a globally recognized standard for assessing the prognosis of NPC, has been criticized as barely satisfactory, as it does not account for important prognostic factors, such as Epstein–Barr virus (EBV) DNA and serum lactate dehydrogenase (LDH) levels (11–13). Therefore, this study aimed to evaluate several potential prognostic factors and construct a prognostic score to classify risk status and identify suitable patients with stage III-IVa NPC that could benefit from IC, using data from a large cohort of patients.

## Materials and Methods

### Patients

**Figure 1:**
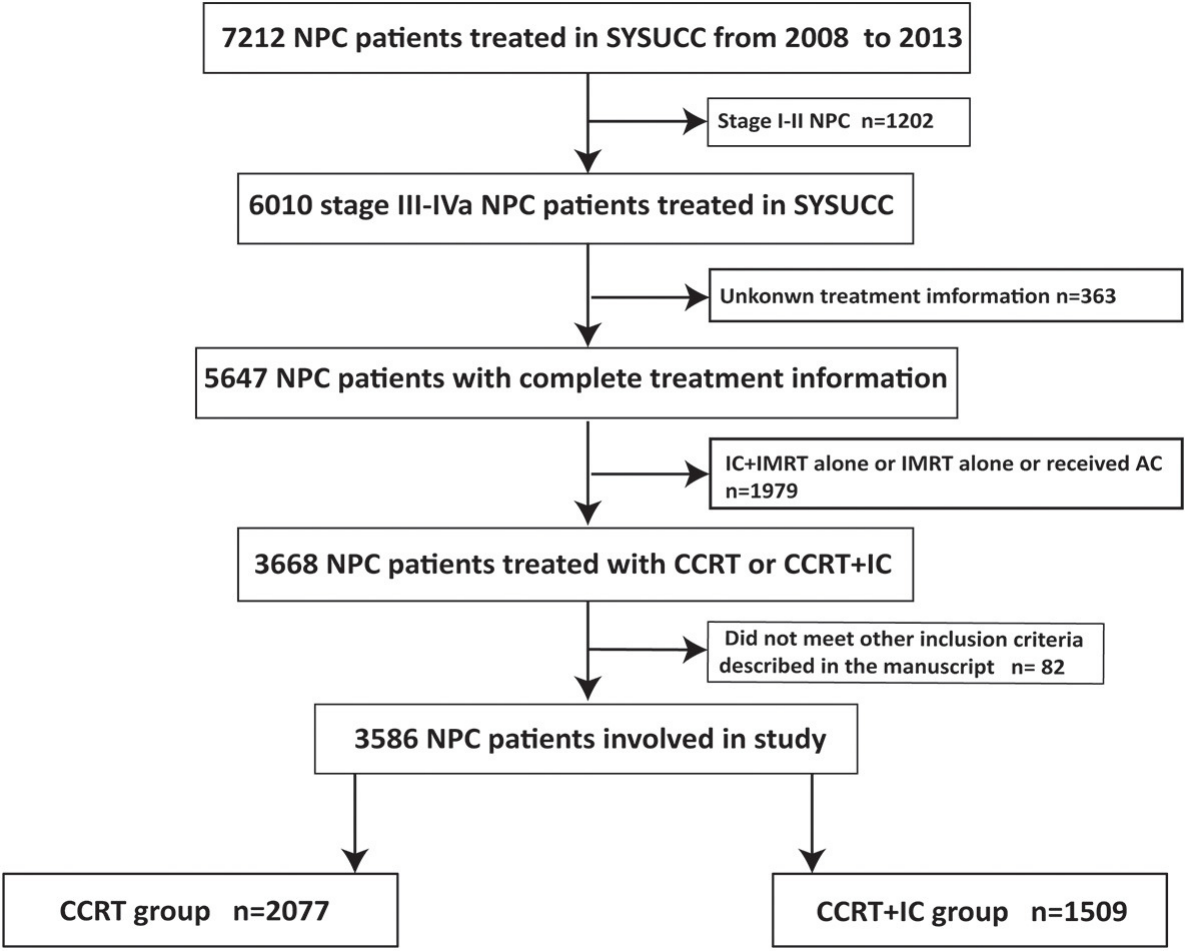
Flowchart presenting patient inclusion process.

This study included 3,586 stage III-IVa NPC patients treated at Sun Yat-sen University Cancer Center (SYSUCC) from January 2008 to December 2013 who met the following inclusion criteria: (1) biopsy-confirmed NPC; (2) non-metastasis status at diagnosis; (3) stage III-IVa NPC, based on the 8th edition of the American Joint Committee on Cancer/International Union Against Cancer (AJCC/UICC) staging system; (4) no history of malignancy or synchronous cancer; (5) complete clinicopathological and treatment information; (6) treatment with IC + CCRT or CCRT alone. The study protocol was approved by the clinical research ethics committee of our cancer center, and written informed consent was obtained from each patient. The flowchart capturing patient inclusion process is shown in Figure 1.

### Quantification of Plasma EBV DNA Levels

Plasma EBV DNA quantification method is described in the Supplementary Material.

### Treatment

In our cohort, 2077 patients were treated with CCRT alone and 1509 patients were treated with IC before CCRT. The common IC regimens were cisplatin (80 mg/m^2^) with 5-fluorouracil (800 mg/m^2^/day over 120 h), or cisplatin (80 mg/m^2^) with docetaxel (80 mg/m^2^), or cisplatin (60 mg/m^2^) with 5-fluorouracil (600 mg/m^2^ over 120 h), and docetaxel (60 mg/m^2^) administered at 3-week intervals. Concurrent chemotherapy consisted of cisplatin/nedaplatin (80 or 100 mg/m^2^) given in Week 1, 4, and 7 of radiotherapy, or cisplatin/nedaplatin (40 mg/m^2^) given weekly during radiotherapy, beginning on the first day of radiotherapy. The radiotherapy technique used was intensity-modulated radiotherapy (IMRT), with 66–70 Gy to the primary lesion, 60–70 Gy to the involved neck fields, and 50–54 Gy of prophylactic irradiation to the neck. All patients received 5 fractions per week at a dose of 1.8–2.2 Gy per fraction. The IMRT plan was designed based on evidence from previous studies (14).

### Follow-Up

All patients received comprehensive follow-up examinations every 3 months for the first 3 years and every 6 months thereafter. Follow-up examinations included semiannual quantitative EBV DNA determination, nasopharyngoscopy, head and neck magnetic resonance imaging, chest radiography, and abdominal sonography. If locoregional relapse and/or distant metastasis were suspected, a bone scan, or 18F-fluorodeoxyglucose positron emission tomography and computed tomography (PET/CT) were considered.

The primary endpoint of the present study was progression-free survival (PFS), which represented the time interval between first diagnosis and disease progression or death from any cause. The following survival outcomes were secondary study endpoints: overall survival (OS) was defined as the time interval from the first diagnosis to death from any cause, while locoregional relapse–free survival (LRFS) and distant metastasis-free survival (DMFS) were defined as time from diagnosis to disease relapse in the nasopharynx or a neck lymph node, and to occurrence of distant metastasis, respectively. Patients lost to follow-up or alive without distant metastasis or locoregional recurrence at the last follow-up visit had their data censored.

### Statistical Analysis

The patients' clinical characteristics and acute toxicity status were compared between treatment groups using the Pearson χ^2^-test or Fisher's exact test. Kaplan-Meier curves were used to compare survival outcomes between study groups with a log-rank test. All variables were transformed into categorical variables. A Cox proportional hazards model with a backward method was used for multivariate analyses. Covariates that were statistically significant (*P* < 0.05) were selected to construct the PI. The cohort was divided into low- and high-risk subgroups by the cutoff value of the PI score, which was determined by a receiver operating characteristic (ROC) curve. All statistical analyses were conducted using SPSS v23 (IBM, Armonk, IL, USA).

## Results

### Patients' Characteristics and Survival

From January 2008 to December 2013, 3586 patients were involved in this study. Then median age of our cohort at diagnosis was 46 years; 74.7% of patients were men. In total, 1509 patients (42.1%) received IC before CCRT. Patient characteristics by treatment group are shown in Table 1. Patients with stage T4 or N2-3 disease were more likely to receive IC than were patients with stage T1-3 (*P* < 0.001) or N0-1 (*P* < 0.001) disease. Male gender (*P* = 0.036), higher (>245 U/L) level of LDH (*P* = 0.001), and pretreatment EBV DNA >1,500 copies/ml (*P* < 0.001) were also significantly associated with IC treatment. During a median follow-up time of 44.9 months (interquartile range 32.8–61.9 months), 299 patients (8.3%) died. The OS rates at 3 and 5 years were 94.0 and 88.9%, respectively.

**Table 1:** Baseline characteristics of patients in the CCRT and CCRT + IC groups.

**Characteristic**	**CCRT *n* (%)**	**CCRT + IC *n* (%)**	***P-*value**
**Total**	2,077	1,509	
**Age, y**			
≤ 46	1,009 (48.6)	769 (51.0)	0.166
>46	1,068 (51.4)	740 (49.0)	
**Gender**			
Female	552 (26.6)	354 (23.5)	0.036
Male	1,525 (73.4)	1,155 (76.5)	
**Diabetes mellitus**			
No	2,014 (97.0)	1,480 (98.1)	0.042
Yes	63 (3.0)	29 (1.9)	
**Cardiovascular disease**			
No	1,957 (94.2)	1,431 (94.8)	0.459
Yes	120 (5.8)	78 (5.2)	
**T stage^a^**			
T1	69 (3.3)	45 (3.0)	<0.001
T2	232 (11.2)	181 (12.0)	
T3	1,365 (65.7)	740 (49.0)	
T4	411 (19.8)	543 (36.0)	
**N stage^a^**			
N0	328 (15.8)	135 (8.9)	<0.001
N1	759 (36.5)	433 (28.7)	
N2	967 (41.7)	710 (47.1)	
N3	123 (5.9)	231 (15.3)	
**LDH level**			
≤ 245 U/L	1,981 (95.4)	1,399 (92.7)	0.001
>245 U/L	96 (4.6)	110 (7.3)	
**EBV DNA level**			
≤ 1,500 copies/ml	913 (44.0)	425 (28.2)	<0.001
>1,500 copies/ml	1,164 (56.0)	1,084 (71.8)	

*CCRT, concurrent chemoradiotherapy; IC, induction chemotherapy; LDH, lactate dehydrogenase; EBV, Epstein–Barr virus*.

a *According to the 8th edition of the UICC/AJCC staging system*.

*P-values were calculated by a χ^2^-test*.

### Prognostic Factors and Establishment of the Prognostic Index

In multivariate analysis, gender [hazard ratio (HR) = 1.369; 95% confidence interval (CI) = 1.113–1.684; *P* = 0.003], T stage (HR = 1.441; 95% CI = 1.200–1.729; *P* < 0.001), N stage (N2 vs. N0-1: HR = 1.375; 95% CI = 1.138–1.661; *P* = 0.001; N3 vs. N0-1: HR = 1.925; 95% CI = 1.468–2.525; *P* < 0.001), LDH level (HR = 1.414; 95% CI = 1.055–1.897; *P* = 0.021), and EBV DNA level (HR = 2.115; 95% CI = 1.706–2.621; *P* < 0.001) emerged as independent prognostic factors for PFS (Table 2). Subsequently, the PI was constructed based on weighting (derived by the log [adjusted HR]) of these five prognostic factors (Table 3). The results of multivariate analysis in terms of OS, LRFS, and DMFS are also shown in Table 2.

**Table 2:** Multivariable analysis of prognostic factors for progression-free survival, overall survival, locoregional relapse–free survival, and distant metastasis–free survival.

**Characteristic**	**HR**	**95%CI**	***P* value**
**Progression-free survival**			
Gender	1.369	1.113–1.684	0.003
T stage	1.441	1.200–1.729	<0.001
N stage			
N2 vs. N0-1	1.375	1.138–1.661	0.001
N3 vs. N0-1	1.925	1.468–2.525	<0.001
LDH level	1.414	1.055–1.897	0.021
EBV-DNA level	2.115	1.706–2.621	<0.001
Treatment method	0.761	0.640–0.905	0.002
**Overall survival**			
Age	1.447	1.146–1.827	0.002
Gender	1.993	1.442–2.755	<0.001
T stage	1.648	1.286–2.111	<0.001
N stage			
N2 vs. N0-1	1.679	1.290–2.187	<0.001
N3 vs. N0-1	2.468	1.692–3.599	<0.001
EBV-DNA level	2.330	1.703–3.189	<0.001
Treatment method	0.552	0.431–0.706	<0.001
**Locoregional relapse–free survival**			
T stage	1.544	1.151–2.072	0.004
EBV-DNA level	2.102	1.486–2.971	<0.001
**Distant metastasis–free survival**			
Gender	1.754	1.336–2.302	<0.001
Diabetes mellitus	1.622	0.982–2.680	0.059
T stage	1.360	1.088–1.701	0.007
N stage			
N2 vs. N0-1	1.434	1.134–1.813	0.003
N3 vs. N0-1	2.491	1.818–3.413	<0.001
EBV-DNA level	1.435	1.014–2.031	0.041
LDH level	2.378	1.813–3.119	<0.001
Treatment method	0.651	0.526–0.805	<0.001

*HR, hazard ratio; CI, confidence interval; NPC, nasopharyngeal carcinoma; LDH, lactate dehydrogenase; EBV, Epstein–Barr virus. A Cox proportional hazards model was used to perform multivariate analyses. All variables were transformed into categorical variables. HRs were calculated for age (years) (>46 vs. ≤ 46), gender (male vs. female), diabetes mellitus (yes vs. no), cardiovascular disease (yes vs. no), T stage (T4 vs. T1-3), LDH level (>245 U/L vs. ≤ 245 U/L), EBV DNA level (>1,500 copies/ml vs. ≤ 1,500 copies/ml) and treatment method (CCRT + IC vs. CCRT). We selected variables using a backward stepwise approach. The P-value threshold was 0.1 (P > 0.1) for removing non-significant variables from the model*.

**Table 3:** Prognostic score to predict progression-free survival.

**Variable**	**Hazard ratio**	**Score [HR = exp (score)]**
**Gender**		
Female	1	0
Male	1.369	0.314
**T stage^a^**		
T1-3	1	0
T4	1.441	0.365
**N stage^a^**		
N0-1	1	0
N2	1.375	0.318
N3	1.925	0.655
**LDH level**		
≤ 245 U/L	1	0
>245 U/L	1.414	0.346
**EBV-DNA level**		
≤ 1500 copies/ml	1	0
>1500 copies/ml	2.115	0.749

*Hazard ratios were estimated by a Cox proportional hazards regression*.

a *According to the 8th edition of the UICC/AJCC staging system*.

### Risk Stratification

**Figure 2:**
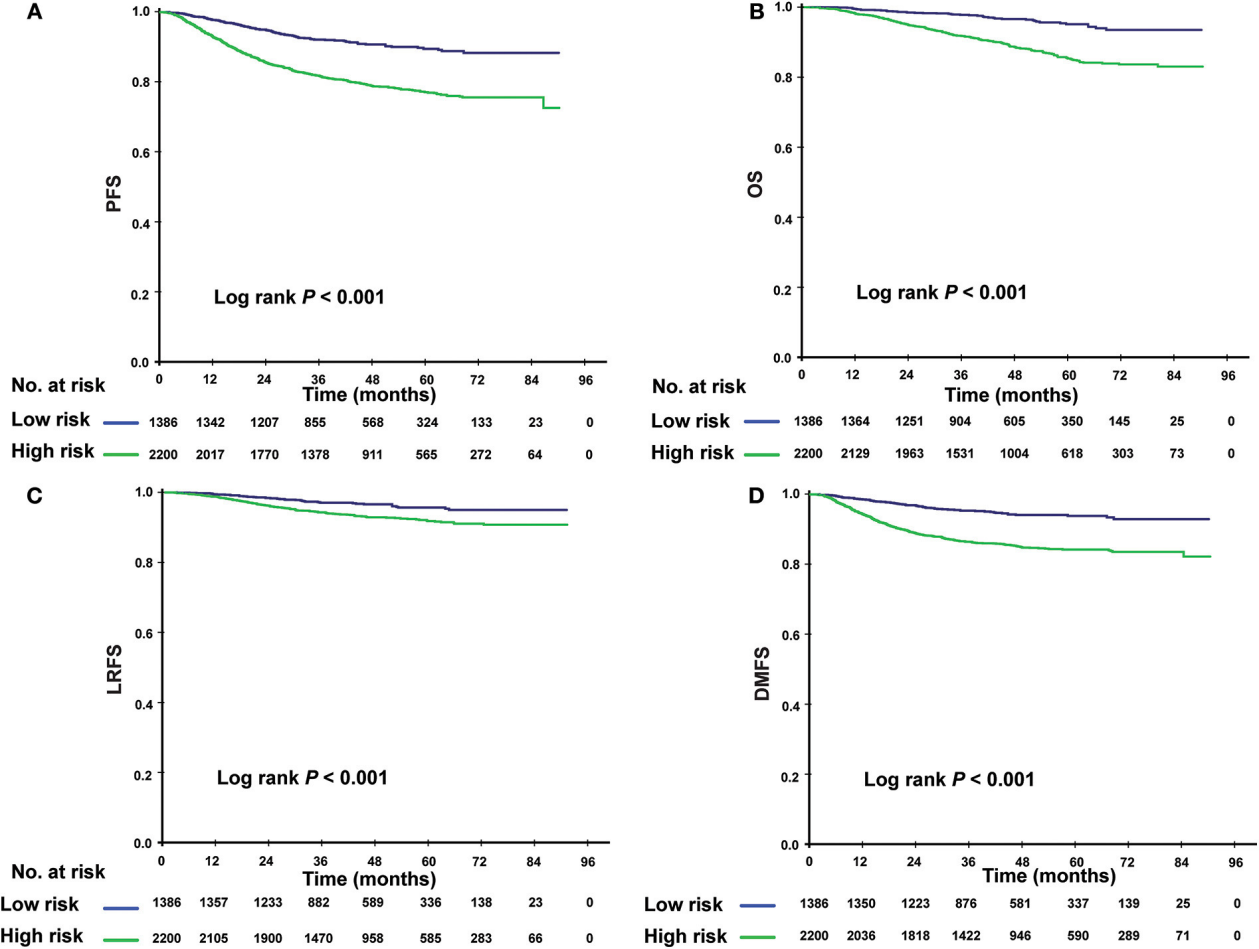
Kaplan-Meier survival curves for the low- and high-risk subgroups. Results shown are for progression-free survival **(A)**, overall survival **(B)**, locoregional relapse–free survival **(C)**, and distant metastasis-free survival **(D)**. *P*-values are calculated using the log-rank test.

Using this PI model, we divided patients into low- and high-risk subgroups. The cutoff value was PI = 0.8, determined by the ROC analysis. Clinical characteristics of patients in two risk subgroups are shown in Table 4. Patients with lower PI (low-risk) achieved a significantly greater PFS compared with high-risk patients (3-year PFS rate: 92.1vs. 81.7%; *P* < 0.001). A similar association was found for OS, LRFS, and DMFS (3-year OS rate: 97.8 vs. 91.7%; *P* < 0.001; 3-year LRFS rate: 97.1 vs. 94.2%; *P* < 0.001; 3-year DMFS rate: 95.3 vs. 86.5%; *P* < 0.001; Figures 2A–D).

**Table 4:** Clinical characteristics of patients in low- and high-risk subgroups.

	**Low-risk patients** ***n*** **(%)**			**High-risk patients** ***n*** **(%)**		
**Characteristic**	**CCRT**	**CCRT + IC**	***P-value***	**CCRT**	**CCRT + IC**	***P-value***
**Total**	972	414		1,105	1,095	
**Age, y**						
≤ 46	503 (51.7)	224 (54.1)	0.445	506 (45.8)	545 (49.8)	0.066
>46	469 (48.3)	190 (45.9)		599 (54.2)	550 (50.2)	
**Gender**						
Female	357 (36.7)	143 (34.5)	0.464	195 (17.6)	211 (19.3)	0.350
Male	615 (63.3)	271 (65.5)		910 (82.4)	884 (80.7)	
**Diabetes mellitus**						
No	943 (97.0)	408 (98.6)	0.097	1,071 (96.9)	1,072 (97.9)	0.179
Yes	29 (3.0)	6 (1.4)		34 (3.1)	23 (2.1)	
**Cardiovascular disease**						
No	911 (93.7)	400 (96.6)	0.028	1,046 (94.7)	1,031 (94.2)	0.643
Yes	61 (6.3)	14 (3.4)		59 (5.3)	64 (5.8)	
**T stage^a^**						
T1	26 (2.7)	6 (1.4)	<0.001	43 (3.9)	39 (3.6)	<0.001
T2	69 (7.1)	40 (9.7)		163 (14.8)	141 (12.9)	
T3	762 (78.4)	247 (59.7)		603 (54.6)	493 (45.0)	
T4	115 (11.8)	121 (29.2)		296 (26.8)	422 (38.5)	
**N stage^a^**						
N0	262 (27.0)	77 (18.6)	0.005	66 (6.0)	58 (5.3)	<0.001
N1	456 (46.9)	204 (49.3)		303 (27.4)	229 (20.9)	
N2	241 (24.8)	125 (30.2)		626 (56.7)	585 (53.4)	
N3	13 (1.3)	8 (1.9)		110 (10.0)	223 (20.4)	
**LDH level**						
≤ 245 U/L	957 (98.5)	410 (99.0)	0.462	1024 (92.7)	989 (90.3)	0.056
>245 U/L	15 (1.5)	4 (1.0)		81 (7.3)	106 (9.7)	
**EBV DNA level**						
≤ 1,500 copies/ml	880 (90.5)	371 (89.6)	0.621	33 (3.0)	54 (4.9)	0.021
>1,500 copies/ml	92 (9.5)	43 (10.4)		1,072 (97.0)	1,041 (95.1)	

*CCRT, concurrent chemoradiotherapy; IC, induction chemotherapy; LDH, lactate dehydrogenase; EBV, Epstein–Barr virus*.

a *According to the 8th edition of the UICC/AJCC staging system*.

*P-values were calculated by a χ^2^-test*.

### The Efficacy of IC in Risk-Based Subgroups

**Figure 3:**
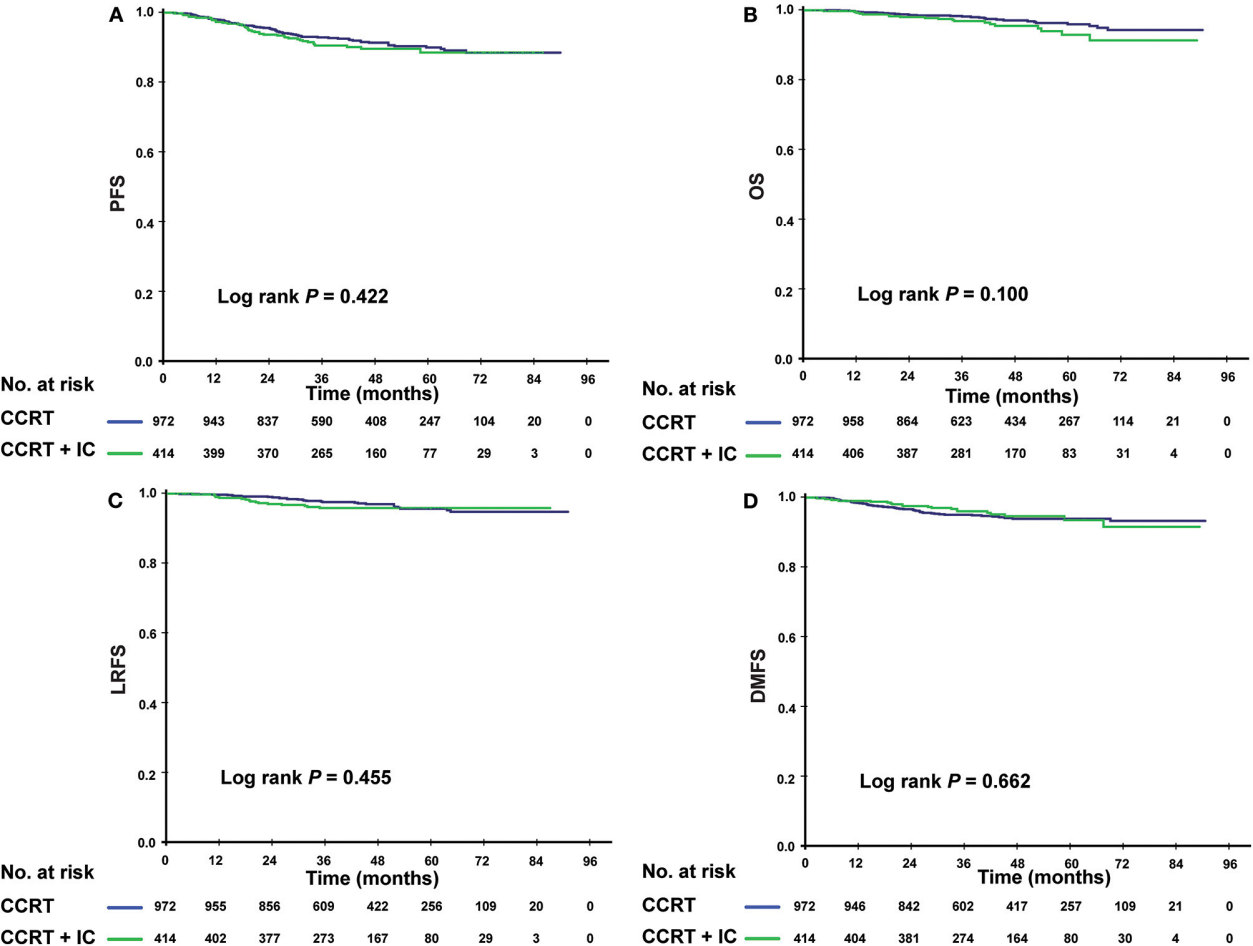
Kaplan-Meier survival curves for the patients receiving concurrent chemoradiotherapy (CCRT), and induction chemotherapy combined with concurrent chemoradiotherapy (IC + CCRT) within the low-risk subgroup. Results shown are for progression-free survival **(A)**, overall survival **(B)**, locoregional relapse-free survival **(C)**, and distant metastasis–free survival **(D)**. *P*-values are calculated using the log-rank test.

**Figure 4:**
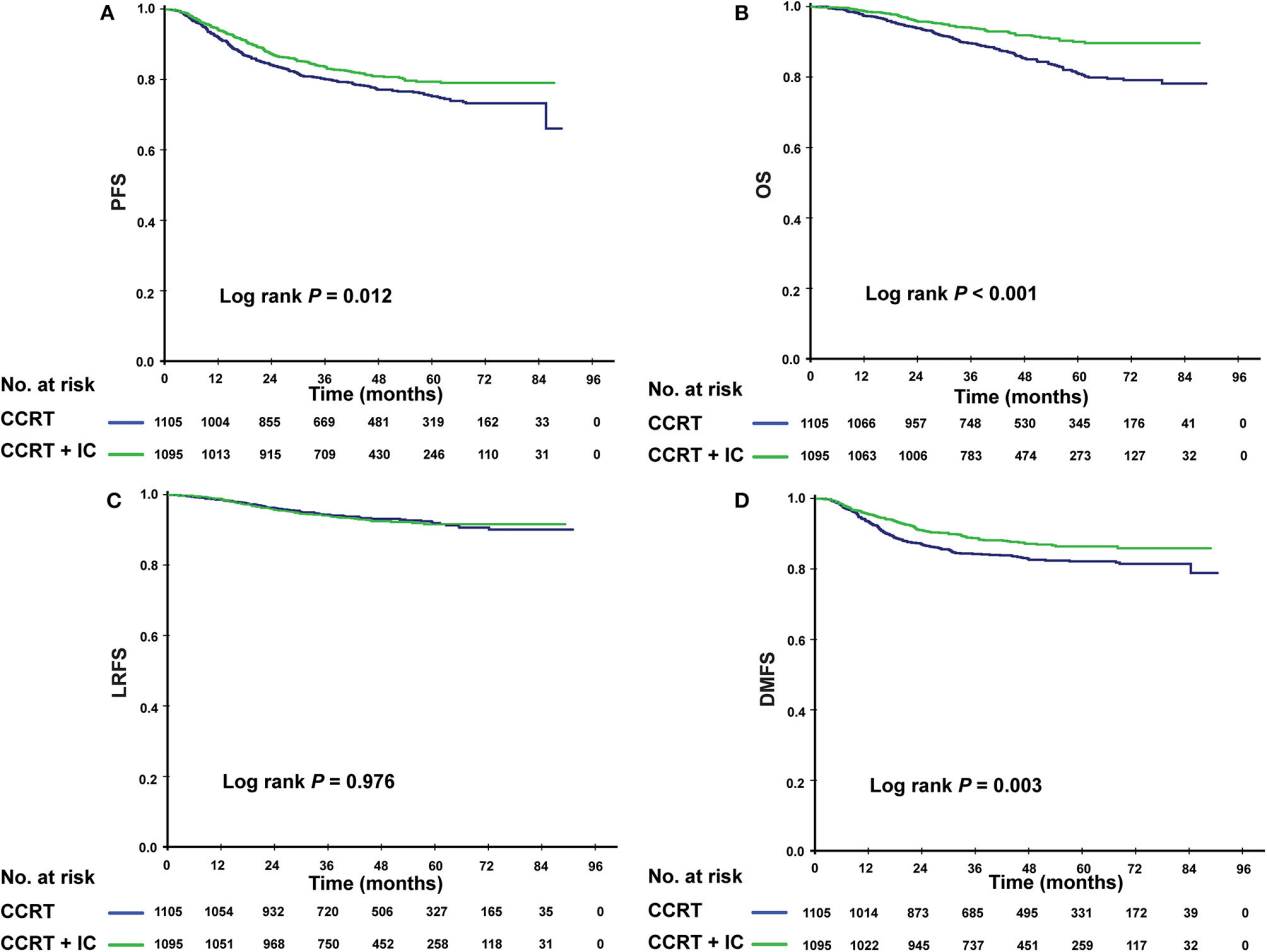
Kaplan–Meier survival curves for the patients receiving concurrent chemoradiotherapy (CCRT), and induction chemotherapy combined with concurrent chemoradiotherapy (IC + CCRT) within the high-risk subgroup. Results shown are for progression-free survival **(A)**, overall survival **(B)**, locoregional relapse-free survival **(C)**, and distant metastasis-free survival **(D)**. *P*-values are calculated using the log-rank test.

Given that patients in different risk subgroups were likely to suffer different tumor burden, we investigated the efficacy of IC in low- and high-risk patients and found that it differed between the subgroups. In the low-risk subgroup (PI <0.8), non-significant differences were observed in PFS (*P* = 0.422), OS (*P* = 0.100), LRFS (*P* = 0.455), and DMFS (*P* = 0.662) between the IC + CCRT and CCRT groups (Figures 3A–D). However, in the high-risk subgroup, patients receiving IC + CCRT achieved greater PFS, OS, and DMFS than did patients receiving CCRT alone (3-year PFS rate: 83.5 vs. 77.9%, *P* = 0.012; 3-year OS rate: 94.0 vs. 89.4%, *P* < 0.001; 3-year DMFS rate: 88.6 vs. 84.2%, *P* = 0.003). There was no significant difference between two treatment groups in LRFS (Figures 4A–D).

In multivariate analysis, within the low-risk subgroup, there was no significant survival difference between two treatment groups (*P* > 0.05 for all survival endpoints). In contrast, in the high-risk subgroup, the addition of IC was found to be protective for PFS (HR = 0.709; 95% CI, 0.585–0.858; *P* = 0.002), OS (HR = 0.466; 95% CI = 0.356–0.610; *P* < 0.001), and DMFS (HR = 0.633; 95% CI = 0.502–0.797; *P* < 0.001). There was no effect for LRFS (Table 5).

**Table 5:** Multivariate analysis of progression-free survival, overall survival, locoregional relapse–free survival, and distant metastasis–free survival in low- and high-risk subgroups.

**Low-risk subgroup**				**High-risk subgroup**			
**Characteristic**	**HR**	**95% CI**	***P-*value**	**Characteristic**	**HR**	**95% CI**	***P-*value**
**Progression-free survival**							
Gender	1.833	1.083–3.103	0.024	Gender	1.397	1.080–1.807	0.011
T stage	1.545	0.953–2.504	0.077	T stage	1.427	1.164–1.749	0.001
N stage				N stage			
N1 vs. N0	1.079	0.672–1.731	0.754	N1 vs. N0	1.486	1.182–1.867	0.001
N2 vs. N0	6.441	2.307–17.988	<0.001	N2 vs. N0	1.938	1.428–2.631	<0.001
EBV DNA level	3.603	1.806–7.189	<0.001	LDH level	1.437	1.066–1.936	0.017
				EBV DNA level	1.556	0.920–2.630	0.099
				Treatment method	0.709	0.585–0.858	<0.001
**Overall survival**							
Age	1.810	0.993–3.299	0.053	Age	1.351	1.048–1.741	0.020
Gender	5.075	1.557–16.537	0.007	Gender	1.774	1.222–2.577	0.003
Cardiovascular disease	2.787	1.22–6.365	0.015	T stage	1.511	1.156–1.975	0.003
T stage	2.382	1.135–5.001	0.022	N stage			
N stage				N1 vs. N0	1.657	1.222–2.246	0.001
N1 vs. N0	1.652	0.785–3.476	0.186	N2 vs. N0	2.310	1.537–3.472	<0.001
N2 vs. N0	11.017	1.082–112.183	0.043	Treatment method	0.466	0.356–0.610	<0.001
EBV DNA level	9.119	2.154–38.612	0.003				
**Locoregional relapse–free survival**							
T stage	1.961	0.990–3.887	0.054	T stage	1.431	1.028–1.991	0.034
EBV DNA level	2.331	1.055–5.149	0.036				
**Distant metastasis–free survival**							
Gender	2.382	1.126–5.039	0.023	Gender	1.840	1.309–2.586	<0.001
N stage				Diabetes mellitus	1.791	1.063–3.015	0.028
N2 vs. N0-1	1.072	0.606–1.897	0.812	T stage	1.295	1.015–1.651	0.037
N3 vs. N0-1	7.237	1.888–27.739	0.004	N stage			
EBV-DNA level	4.371	1.769–10.801	0.001	N2 vs. N0-1	1.506	1.141–1.988	0.004
				N3 vs. N0-1	2.366	1.667–3.358	<0.001
				LDH level	1.425	1.001–2.028	0.050
				Treatment method	0.633	0.502–0.797	<0.001

*HR, hazard ratio; CI, confidence interval; LDH, lactate dehydrogenase; EBV, Epstein–Barr virus; CCRT, concurrent chemoradiotherapy; IC, induction chemotherapy*.

*A Cox proportional hazards model was used to perform multivariate analyses. All variables were transformed into categorical variables. HRs were calculated for age (years) (>46 vs. ≤ 46), gender (male vs. female), diabetes mellitus (yes vs. no), cardiovascular disease (yes vs. no), T stage (T4 vs. T1-3), LDH level (>245 U/L vs. ≤ 245 U/L), EBV DNA level (>1,500 copies/ml vs. ≤ 1,500 copies/ml) and treatment method (CCRT + IC vs. CCRT)*.

*We selected variables with a backward stepwise approach. The P-value threshold was 0.1 (P > 0.1) for removing non-significant variables from the model*.

### Acute Toxicity

Details of treatment-related acute toxicity experienced by patients receiving CCRT and CCRT + IC are presented in Table 6. The IC + CCRT group had a significantly higher proportion of grade 3–4 leukopenia (30.0 vs. 16.3%; *P* < 0.001) and neutropenia (37.8 vs. 15.2%; *P* < 0.001) than did the CCRT alone group. Between-group differences in other hematological toxicities, such as anemia or thrombocytopenia, were not significant. No significant differences in grade 3–4 hepatotoxicity or nephrotoxicity were observed between the treatment groups.

**Table 6:** Acute toxicities in patients between CCRT and CCRT + IC groups.

**Adverse event (Toxicity grade)**	**CCRT (*****n*** **=** **2,077)**		**CCRT** **+** **IC (*****n*** **=** **1,509)**		***P-*value**
	**0–2 (%)**	**3–4 (%)**	**0–2 (%)**	**3–4 (%)**	
Leukocytopenia	1,738 (83.7)	339 (16.3)	1,056 (70.0)	453 (30.0)	<0.001
Neutropenia	1,761 (84.8)	316 (15.2)	939 (62.2)	570 (37.8)	<0.001
Anemia	2,033 (97.9)	44 (2.1)	1,481 (98.2)	28 (1.8)	0.631
Thrombocytopenia	2,052 (98.8)	25 (1.2)	1,484 (98.3)	25 (1.7)	0.313
Hepatoxicity	2,054 (98.9)	23 (1.1)	1,482 (98.2)	27 (1.8)	0.112
Nephrotoxicity	2,077 (100.0)	0 (0.0)	1,507 (99.9)	2 (0.1)	0.177^a^

*CCRT, concurrent chemoradiotherapy; IC, induction chemotherapy*.

a *P-value was calculated with the Pearson χ^2^-test or Fisher's exact test*.

## Discussion

The present study identified independent prognostic factors for patients with stage III-IVa NPC in the IMRT era. Our study involved a large cohort and development of a PI to personalize treatment recommendations for IC. For patient stratification, the PI cutoff value was determined by the ROC analysis. We found that high-risk patients are likely to benefit from the addition of IC before CCRT, whereas low-risk patients are unlikely to benefit from it.

CCRT is standard treatment for locoregionally advanced NPC. As radiotherapy technology has developed, the local control rate of NPC has improved significantly (15). In the IMRT era, occurrence of distant metastasis has become the predominant sign of failed treatment (16, 17). Recently, several clinical trials have provided evidence that IC before definitive CCRT is associated with lower incidence of distant metastases and further improved patient survival (7, 18, 19). However, according to studies among patients with stage T3-4 N0-1, clinical outcome was similar between the CCRT and IC + CCRT groups, indicating that IC might benefit only patients with a greater tumor burden (9, 10). Considering the toxicity and economic cost of chemotherapy, it is important to identify suitable patients who could benefit from additional IC.

The current AJCC/UICC stage classification is the main guideline for NPC risk stratification in clinical practice. However, this classification does not consider several variables that have been suggested as prognostic factors in NPC, such as age, gender, LDH, comorbidities, and, in particular, EBV DNA (11–13). Therefore, a more comprehensive prognostic model is urgently needed to accurately predict patients' clinical outcome.

In previous studies, several prognostic models were put forward to help select high-risk patients that might benefit from IC (20–22). Zhang et al. developed and validated a nomogram to predict individual benefit of IC based on a Phase III clinical trial (22). However, only 480 participants were involved in the establishment of the nomogram, and plasma EBV DNA was not included. Similarly, Du et al. created a prognostic model for distant metastasis in locally advanced NPC patients to identify high-risk patients who should receive IC, where the prognostic score was the sum of the number of prognostic factors (20). It was less rigorous in that different risk factors did not share the same weight in treatment failure. In our study, the PI scores were calculated based on the logarithm of HRs derived from multivariate analysis. To our knowledge, to-date, our study involved the largest cohort in establishing prognostic scores for selecting candidates for IC in stage III-IVa NPC.

We set PFS as the primary endpoint. In our results, five characteristics (gender, T stage, N stage, LDH level, and EBV DNA level) were selected and remained independent factors in multivariate analysis. Previous studies have verified all five of these factors as important prognostic indicators (11). After the trade-off between sensitivity and specificity was resolved, the cutoff value of PI was determined as 0.8, with 1386 and 2200 patients identified as at low- and high-risk, respectively. Among patients with a higher PI, patients achieved a higher PFS rate, if they were in the IC + CCRT group. In contrast, no significant differences between the treatment groups were observed in the low-risk subgroup.

Collectively, our findings justify the recommendation of IC for patients identified as at high-risk, which could be explained by the following reasons. IC plays an important role in early eradication of tumor before radical radiotherapy. Patients in the high-risk subgroup suffer a greater tumor burden and higher risk of treatment failure. Some of them also might develop subclinical micrometastasis at diagnosis, which might indicate they should receive intensified therapy. Concurrently, induction chemotherapy can reduce tumor volume, which can support radiotherapy and shrink the target area. As a result, long-term toxicities, such as radiation encephalopathy, xerostomia, or trismus might improve to some extent. Therefore, the addition of IC could help these patients achieve longer disease-free survival. However, patients in the low-risk subgroup had a relatively satisfactory clinical outcome when treated with CCRT alone. Concurrently, additional toxic effects such as hepatoxicity or nephrotoxicity caused by IC may influence the survival benefit.

Our study shows great potential for application in clinical practice. Clinicians could evaluate the condition of stage III-IVa NPC patients before treatment using our PI system and select high-risk patients who may benefit from IC. However, it should be noted that more than 10% of high-risk patients still developed distant lesions in the IC + CCRT group, suggesting that a more intense therapy such as targeted therapy may be necessary for this subgroup (23, 24). Our group has launched a phase 1 study of tumor-infiltrating lymphocyte immunotherapy after CCRT in high-risk NPC; we were looking forward to the results of the phase 2 study (25).

Although this study is based on a large cohort, it has several limitations. First, it was a retrospective study, so survival outcomes might be affected by confounding factors, and accurate data on late toxicities could not be acquired. Secondly, the data were obtained from a single treatment center; therefore, our results should be validated by other datasets.

In conclusion, we proposed a PI model to predict whether patients could benefit from additional IC before CCRT and thereby to improve the decision-making process for patients with stage III-IVa NPC. Patients with higher PI (>0.8) are identified as at high-risk and would be likely to benefit from additional IC, whereas low-risk patients are unlikely to benefit from it.

## Data Availability Statement

The datasets analyzed in this article are not publicly available. Requests to access the datasets should be directed to maihq@mail.sysu.edu.cn.

## Ethics Statement

This retrospective study was approved by the Clinical Research Committee of SYSUCC. Patients were required to provide written informed consent before enrolling in the study.

## Author Contributions

H-QM, L-QT, Q-YC, and LY: study concepts and manuscript review. X-SS, B-BX, Z-JL, and S-LL: study design, data acquisition, and data analysis and interpretation. X-SS, B-BX, and Z-JL: quality control of data and algorithms, statistical analysis, and manuscript preparation and editing.

### Conflict of Interest

The authors declare that the research was conducted in the absence of any commercial or financial relationships that could be construed as a potential conflict of interest. The handling Editor declared a shared affiliation, though no other collaboration, with the authors.
