# Supplementary material for: Stratification of Candidates for Induction Chemotherapy in Stage III-IV Nasopharyngeal Carcinoma: A Large Cohort Study Based on a Comprehensive Prognostic Model
Source: Front Oncol. 2020 Feb 28;10:255. doi: 10.3389/fonc.2020.00255 (PMC7059214; doi:10.3389/fonc.2020.00255)
Supplement: Supplementary file 1 [file Data_Sheet_1.DOCX]

The detailed processes are as follows:

DNA Extraction from Plasma Samples

Samples of peripheral blood (5 mL) were collected in an ethylenediamine tetraacetic acid tube from all participants and were centrifuged at ×1600g for isolation of plasma. Plasma samples were transferred carefully into plain polypropylene tubes for storage at ﹣80 °C until further processing. DNA from plasma samples was extracted with the QIAamp Blood Kit (Qiagen, Hilden, Germany) using the blood and body fluid protocol, as recommended by the manufacturer. In total, 500 –1000µL of each plasma sample were used for DNA extraction per column, with a final elution volume of 50µL from the extraction column. The exact amounts were documented for the calculation of the target DNA concentration.

Real-Time Quantitative EBV DNA PCR

The real-time quantitative PCR system was developed for EBV DNA detection toward the BamHI-W region. The system consisted of the amplification primers W-44F (5’-AGT CTC TGC CTC CAG GCA-3’) and W-119R (5’-ACA GAG GGC CTG TCC ACC G-3’) and the dual-labeled fluorescent probe W-67T (5’- [FAM] CAC TGT CTG TAA AGT CCA GCC TCC [TAMRA]-3’). In this study, real-time quantitative PCR for the β-actin gene was used as a control for the amplifiability of plasma DNA. Theβ-actin gene primer sequence was forward, 5’-ACA GGC ACCA GGG CGT GA TGG-3’; and reverse, 5’-CTC CAT GTC GTC CCA GTT GGT-3’; and the dual-labeled fluorescent probe sequence (5’-[FAM] CAT CCT CAC CCT GAA GTA CCC CAT C [TAMRA]-3’).
